# Prediction and prevention of suicide in patients with unipolar depression and anxiety

**DOI:** 10.1186/1744-859X-6-23

**Published:** 2007-09-05

**Authors:** Xenia Gonda, Konstantinos N Fountoulakis, George Kaprinis, Zoltan Rihmer

**Affiliations:** 1 Clinical Psychologist, Department of Psychiatry, No. III, National Institute for Psychiatry and Neurology, Budapest, Hungary; 2 3rd Department of Psychiatry, Aristotle University of Thessaloniki, Greece; 3 Department of Psychiatry and Psychotherapy, Semmelweis Medical University, Budapest, Hungary

## Abstract

Epidemiological data suggest that between 59 and 87% of suicide victims suffered from major depression while up to 15% of these patients will eventually commit suicide. Male gender, previous suicide attempt(s), comorbid mental disorders, adverse life-situations, acute psycho-social stressors etc. also constitute robust risk factors. Anxiety and minor depression present with a low to moderate increase in suicide risk but anxiety-depression comorbidity increases this risk dramatically Contrary to the traditional psychoanalytic approach which considers suicide as a retrospective murder or an aggression turned in-wards, more recent studies suggest that the motivations to commit suicide may vary and are often too obscure. Neurobiological data suggest that low brain serotonin activity might play a key role along with the tryptophan hydroxylase gene. Social factors include social support networks, religion etc. It is proven that most suicide victims had asked for professional help just before committing suicide, however they were either not diagnosed (particularly males) or the treatment they received was inappropriate or inadequate. The conclusion is that promoting suicide prevention requires the improving of training and skills of both psychiatrists and many non-psychiatrists and especially GPs in recognizing and treating depression and anxiety. A shift of focus of attention is required in primary care to detect potentially suicidal patients presenting with psychological problems. The proper use of antidepressants, after a careful diagnostic evaluation, is important and recent studies suggest that successful acute and long-term antidepressant pharmacotherapy reduces suicide morbidity and mortality.

## Background

Understanding why aggression and destruction becomes directed towards the self is a major challenge for psychiatry, psychology and philosophy as well. Suicide is a complex, multicausal behavioural phenomenon, and to be able to understand the underlying factors a complex approach is required. Although in the past decades there have been unprecedented developments taking place in medicine, with more possibilities to save lives than ever before, we still need more efficient ways to tackle the problem of suicide.

In the past two decades there has been a substantial decline in the suicide rates in most European countries, and also in the US and Canada. The most pronounced decrease took place in countries with traditionally high suicide rates. The decline was greater in women, who more frequently suffer from major depression and also seek medical care more frequently than men do [1-3]. Although the causes of the declining suicide rates are not yet fully understood, research data suggests that better recognition of major depression, as well as better availability of treatment with antidepressants and mood stabilisers (particularly lithium), could be one of the major underlying factors [1,2,4,5].

Therefore, understanding, prediction and prevention of suicidal behaviour is today one of the most challenging tasks in society in general and in psychiatry in particular. It has become a priority in particular during recent years, as several psychological autopsy studies of suicide victims have shown that the majority were suffering from a mood disorder, usually major depression, with frequent comorbidity of various other mental disorders (in particular anxiety disorders [6-8]).

This line of evidence suggests that about 90% of suicide victims suffered at least from one major (Axis I) mental disorder, with major depression being the mental disorder most related to the manifestation of suicidal behaviour [6-11]. According to the most recent psychological autopsy studies that have used current diagnostic classifications and sound methodology, the rate of current major depressive episode among suicide victims from the general population is reported to range between 59% and 87%. What is impressive is the fact that in spite of frequent medical contact before the suicide event, only a small minority of depressive suicide victims had received appropriate antidepressant pharmacotherapy, and this observation is particularly strong concerning primary care [3,6,7,12,13]. An estimated 15% of patients with severe major depression eventually die from suicide. In psychogeriatric populations it has been reported that close to 10% of patients with late-life depression die by their own hands every year [14].

## Factors underlying suicidal behaviour

The psychopathological background of suicide emphasises the role of several factors associated with depression. Psychoanalytic theory emphasises aggression turned inwards and considers suicide equal to a retrospective murder. According to this, motivation for suicide can arise from destructive drives (wish to kill or to be killed) as well as a wish for reunion with someone lost. However, modern psychodynamic and cognitive theories do not consider that suicide victims necessarily possess or manifest a similar psychological or personality structure. Modern approaches tend to focus rather on hopelessness as a core element of suicide, resulting from continuous frustration arising from rigidly held unrealistic expectations, where as a consequence suicide remains the only way out [15]. Accordingly, research with elderly depressive patients with moderate to severe depression suggests that it is more likely for these patients to have suicidal ideation with increasing hopelessness. However, in contrast to these results, research on patients with milder forms of depression suggests that hopelessness seems to have little effect on the extent of suicidal ideation [16]. Although according to common sense, hopelessness could lead to depression, this does not seem to hold true when controlling for the severity of depression. Patients who report moderate or severe depression are more likely to have suicidal ideation with increasing hopelessness, whereas hopelessness *per se *seems to have little effect on the level of ideation at mild or lower depression levels [16]. In other words, hopelessness, guilt and related suicidal behaviour in MD is a state-related, severity-dependent phenomenon, and recurrence of suicidal ideation across depressive episodes shows a high consistency [17-19]. To further complicate things, there are reports correlating suicide with hopelessness also in dysthymia [20], while according to one study it is associated with alexythymia in cases of panic disorder [21].

Suicidal ideation and thinking of death is nonetheless a common feature of the thinking of depressives both before and after a suicide attempt. Thoughts concerning suicide are neither simple nor concrete and are varied in their manifestation. Several patients fear that they will die and do not wish to, while others desire death and are determined to kill themselves. It is not clear whether these states constitute consecutive phases, or represent distinct symptoms and reflect a qualitatively different underlying psychopathology [22]. There are different factors associated with suicide depending on gender, which may also point to different psychological mechanisms in the background of suicide. While in the case of men low social and family support and depersonalisation is related to suicide, in the case of women depressive mood and anxiety is more strongly associated [23].

Identifying biological correlates of suicide is another main target of research. Low brain serotonergic function has long been implicated in the background of aggression and suicide, although low central serotonergic activity is characteristic of depression as well. To date, no biological marker has been found to distinguish explicitly between suicidal and non-suicidal depressives, which suggests that other clinical (such as severity of depression), personality (such as impulsiveness) or psychosocial (acute stressors, low social support, isolation) factors probably also play an important role. Research indicates that low cerebrospinal fluid (CSF) 5-hydroxyindoleacetic acid (5HIAA) concentration might have some predictive value [24,25]. Suicide-attempting depressives have also been found to have lower CSF homovanillic acid (HVA) levels compared to both non-suicide attempting depressives and controls. By contrast, controls had the same CSF HVA concentrations with non-suicidal depressives and this result might point to the involvement of dopaminergic abnormalities in suicide but not in depression [26].

While most studies of suicide and mental disorders concentrate on major depression, one of the few studies dealing with dysthymia suggested that platelet monoamine oxidase (MAO) activity was significantly lower in females but not in male dysthymic patients who had attempted suicide [27].

Hyperactivity of the HPA axis, as reflected in abnormal dexamethason suppression test [28] has also been implicated as a risk factor for suicide in major depression, recent large-sample prospective studies, however, suggest that this may be true only in cases of severely ill and previously hospitalised major depressive patients pointing to some other underlying factor [29].

Panic disorder patients with suicidal thoughts were reported to have lower serum total cholesterol and low-density lipoprotein levels than normal control subjects [30]. The implications of such a finding are still unclear, but it is well documented that low serum cholesterol levels are associated with decreased central serotonin synthesis [31].

The level of omega-3-fatty acids (an important contributor to central serotonin synthesis) has been found to be inversely correlated with lifetime prevalence of unipolar and bipolar depression [32] and also seems to be a powerful predictor of future suicidal behaviour in unipolar major depression [33]

Aggression studies have also concluded that the level of emotional arousal is a crucial factor in expressing aggression whether towards the self or towards others. Results indicate that unless a sufficient level of emotional alertness is present, serotonergic activity cannot be linked to aggressive behaviour [34]. This suggests that the problem may lie in the imbalance between behavioural inhibition mediated by the serotonergic system and the level of arousal mediated by cathecholamines and particularly by acethylcholine [35,36]. In other words, the patient may express aggression either towards the self or towards the environment when a lower threshold is present. In addition to serotonin, behavioural inhibition may be regulated by noradrenalin and dopamine, which play a role in the regulation of serotonin release [35]. Research indicates that the above concerns only impulsive physical aggression and not physical aggression in general [37-40]; however, they may also apply to suicidal behaviour mostly in the frame of current major depression, where impulsiveness also plays a role [22,41]. It has also been found that history of serious impulsive aggressive behaviour is related to serotonergic dysregulation. It should also be noted that arousal, mostly in the form of anxiety, is increased in 'minor' mental disorders and comorbidity of anxiety and depression seems to constitute an important risk factor for suicide.

Family history of suicide in first degree relatives is a suicide risk factor in cases of current major depressive patients [6,8] and there is also evidence of familial aggregation of suicide pointing to genetic factors, a finding also confirmed by twin and adoption studies. Genetic research has discovered a possible role of a polymorphism in the TPH1 gene as a risk factor for suicidal behaviour, encoding the enzyme catalysing the rate-limiting step of serotonin synthesis [42]. Some studies also implicate the role of an insertion/deletion polymorphism of the promoter of the serotonin transporter gene (5-HTTLPR) [43].

Because suicide is a multicausal behaviour, as well as biological and psychopathological factors, social and cultural environment also play important roles in the determination and manifestation of suicidal behaviour. Specific social parameters may promote or inhibit the manifestation of suicidal behaviours, as well as modify their expression [6].

Adverse life events may cause important losses in one's life, such as physical losses from poor health condition or burdening physical disease, sensory deficits or cognitive decline, as well as social losses such as the death of a person close to us or loss of work role or income. These losses, when accompanied with chronic stress, may result in social isolation that is turn worsens depression and leads to the appearance of suicidal ideation. Social isolation and poor social networks constitute a problem especially in the case of the elderly [44,45], where severe physical disease such as renal failure or cancer represent an additional major risk factor for a well-planned suicide attempt [46,47]. The rate of males committing suicide is especially high in old age, while inyounger patients being divorced or widowed is more strongly associated with suicide ideation and attempt than other social factors [31,48]. In addition, in countries such as the US where the multicultural composition of society allows for ethnic comparison, a difference in the prevalence of lifetime suicide attempts among different ethnicities has been described, although causes are not clear yet. Migration, socioeconomic status and acculturation are among the suspected factors playing a role behind these differences, the role of major depression, however, is obvious even in this case [49]. In the US, higher lifetime rates of suicide attempt in different ethnic groups was associated with more frequent lifetime rates of major depression [49].

There have also been gender differences described in the case of social factors associated with suicide. Low social or family support has been found to be associated with suicide in men but not in women, while psychological factors such as depersonalisation or anxiety seem to play a more important role in women [23].

## Suicide risk factors and prediction of suicide in unipolar major depression

Not all patients with major depression commit suicide. Several risk factors for suicidal behaviour have been identified and have been classified as primary (such as the presence of psychiatric and medical conditions, severe somatic illness, previous suicide attempts), secondary (adverse life situations and psychosocial risk factors) and tertiary (demographic factors such as male gender and old age) [6,7]. However, their predictive value is far from satisfactory. Suicide risk is highest when primary risk factors are present; the presence of secondary and tertiary suicide risk factors indicate high suicide risk almost exclusively only in the presence of primary risk factors [6,8]. Unfortunately, the association of risk factors and suicide is mainly statistical, as they can only predict individual cases of suicide to a limited extent. Awareness of risk factors, however, is a valuable tool for clinicians in estimating the suicide risk.

Although severity is reported to be one of the strongest correlates of suicide in patients with a depressive episode [8,50], there is no satisfactory definition for severity of depression. Considering only the number of symptoms concludes that melancholia is a more severe form of depression, and there is no difference in quality between melancholic and non-melancholic depressives. Necessity of hospital admission and degree of disability caused are also possible indices of severity. Most of these considerations, however, yield circular reasoning as definition of disability includes specific symptoms such as suicidal ideation, anhedonia or fatigue. Within this framework, it is thus difficult to find specific syndromes or subtypes of depression associated with suicidal ideation, as suicidal ideation itself is in many cases a central component of the definition of subtypes of depression either directly or indirectly [51]. The clinically most important suicide risk factors in unipolar depression [8,48] are listed in Table 1.

**Table 1:** Clinically explorable suicide risk factors in unipolar depression

Prior suicide attempt
Current suicidal ideation, wish to die, few reasons for living
Severe symptomatology (hopelessness, guilt, insomnia, psychotic features)
Agitation/depressive mixed state
Comorbid substance-use, personality disorder, serious somatic illness
Permanent psycho-social stressors
Recent (acute) adverse life situations
Family history of suicide (1st and 2nd degree relatives)
Lack of family/social and medical support

A risk factor only recently discovered and associated with suicide in depressed patients is the emergence of depressive mixed state (three or more simultaneously co-occurring intra-depressive hypomanic symptoms in patients with 'unipolar depression'), which overlaps with agitated depression to a great extent. Depressive mixed state as well as agitation substantially increases the risk of both attempted and committed suicide [1,2,4,8,52]. They seem to be the strongest cross-sectional predictors and the most potent risk factors for suicide. This is very important as many bipolar patients present with a pseudo-unipolar clinical picture for much of their life. Risk factors for suicide in the case of depressed patients include agitation, depressive mixed states (pseudo-unipolar depression), higher number of prior depressive episodes, comorbid anxiety, personality disorders and alcohol dependence, as well as sociodemographic and psycho-social factors such as younger age, being divorced or widowed, and experiencing adverse life-situations that are associated with increased suicidal ideation and higher prevalence of attempts [2,6-8,31,52].

The high prevalence of major depression among suicide victims also indicates that many of them had been treated for major depression preceding or during their suicidal event, although this is not always the case [1,6,13]. Depressed patients can seek and find professional help at a variety of medical settings and structures. There is only limited research data available concerning the prevalence, method and lethality of suicide in relationship to different healthcare settings the patients had sought help from. Data so far indicate that most variation can be attributed to differences in the clinical socio-demographic characteristics of the patient population in the catchment area supported by the given healthcare setting. Differences in available therapeutic methods might also play a role. Furthermore, data indicate that a significantly higher rate of suicidal patients communicate their intent to commit suicide in a psychiatric care setting than in a general medical care one (59% vs 19%). The same ratio is reflected in treatment; in psychiatric care 60% of victims are given antidepressants in contrast to only 16% in general medical care [12].

To summarise the above, in this context, prediction of suicide is not impossible although it still constitutes a difficult task. The statistical fact is that although depression is very closely related to suicide, more than two thirds of depressed patients never attempt suicide and the vast majority of depressives never complete suicide, indicating that other specific (suicide related) and non-specific factors besides major depression must also play a crucial role. As some associations have been found among personal psychiatric histories, characteristics of depression at index episode and suicidal behaviours, the clinical information could serve as a guide for clinicians. Psychotic patients are consistently more likely to apply violent suicide methods, such as use of guns, hanging or jumping from height [53], and thus they also have a higher risk of completed suicide compared to non-psychotic depressives [54]. In spite of theoretical considerations and vagueness of definitions, the overall severity of symptomatology as well as the presence of hopelessness can also serve as predictors for the clinician. In a recent prospective follow-up study of 269 major depressives (most of whom were treated with antidepressants) among patients having suicidal ideation at baseline, the decline in suicidal ideation was predicted by preceding declines in the levels of both depressive symptoms and hopelessness [55]. The presence of mixed symptoms (pseudo-unipolar depression) or agitation substantially increases the risk of attempted and completed suicide [1,2,4,8,52].

## Early identification and management of suicidal behaviour in unipolar major depression

Because nearly 90% of suicide attempters have major depression, and also because a great majority of patients attempting suicide seek professional medical help prior to their suicidal act, early identification of suicidal behaviour is not only possible to a significant degree, but also intervention could make a difference. Recent studies with suicide victims seeking medical help concerning mental problems before committing suicide concluded that the vast majority of them had contacted a general practitioner (GP) concerning their problems a few months prior to their completed suicide. However, data suggest that in the vast majority they were prescribed 'antifatigue agents' (e.g. vitamins) and anxiolytics instead of proper psychotropic medication [56]. So, although early identification is possible, data indicate that recognition, management and treatment of pre-suicidal patients is suboptimal, if not actually poor [57]. The solution may lie in better recognition of signs of approaching suicide and awareness of treatment possibilities. Promoting suicide prevention in major depressive disorder thus requires improving the training and skills of non-psychiatric healthcare professionals, especially GPs, in recognising and treating depression in medical and primary care [12,58,59].

Once the risk of suicidal behaviour is recognised, several possibilities for prevention (treatment) arise. Lithium has been reported to have a robust anti-suicidal effect both in unipolar depression and bipolar disorders in a recent systematic review of 32 trials including more than 3400 patients [5]. Another comprehensive review of 34 studies involving more than 16000 patients showed a 21-fold risk-reduction for attempted and completed suicide in both unipolar or in bipolar patients on long-term lithium therapy [60]. There is, however, some concern that there may be an over-interpretation of data on lithium as compared to other agents despite its obvious superiority over antidepressants in preventing suicide [61]. Also, the relevance of lithium use to prevent recurrence of unipolar depression has not been adequately studied.

Proper treatment of depression in itself significantly reduces the risk for suicide, and antidepressive agents are the only formally approved treatment for major depression [2,4,62]. There is, however, no data from controlled trials to support an anti-suicidal effect for antidepressants, mainly because suicidal patients are usually excluded from randomised clinical drug trials because of ethical considerations. However, common sense and 'uncontrolled' long-term, real-life clinical follow-up studies including the most severe, frequently suicidal unipolar major depressives suggest that antidepressants possess a marked anti-suicidal effect when used in unipolar depressive patients [54,62]. Concomitant use of benzodiazepines in the first few weeks of treatment significantly speeds up the response to antidepressants at least in patients with major depressive disorder [63].

However, despite the obvious role of antidepressants in the background of declining suicide rates, the US Food and Drug Administration recently issued a warning concerning the use of antidepressants in children and adolescents, and possibly in all age groups, because of possible induction of suicidality (thinking and behaviour but not completed suicide) as a result of antidepressant use by juvenile depressives. There are a few cases where antidepressants do indeed raise the risk of suicide or from the very beginning of treatment generate suicidal behaviour; this, however, possibly happens in cases of unrecognised pseudo-unipolar or subthreshold bipolar patients treated as unipolar patients, and thus these suicidal behaviours could be prevented by suitable recognition of bipolarity within depression [2,4]. In the case of these patients (as well as in overt bipolar patients), antidepressant monotherapy may induce not only rapid cycling and switching to mania but also mixed states, characterised by agitation, irritability, hostility and impulsivity, possibly giving rise to suicidal ideation [2,52]. It is important to note that there might be unrecognised pseudo-unipolar or subthreshold bipolar patients [2] among patients suffering from panic disorder, social phobia or dysthymia, the majority of whom later develop major depressive episodes. In clinical practice it is common to diagnose these disorders as comorbid conditions or as the sole current diagnosis in patients suffering from bipolar disorder, especially when it is not the classic bipolar type.

## Suicide and minor depression and anxiety disorders

The most common psychiatric illnesses in the background of suicide are unipolar major depression and schizophrenia, but other minor mental disorders, such as dysthymia and anxiety disorders, have also been found to increase the risk of suicide, although to a lesser extent. Studying the effects of minor disorders on suicidal behaviour, however, is difficult because they are often present as comorbid conditions of major depression [64]. The importance of minor mental disorders in suicidal behaviour was also demonstrated by two recent studies showing that a pre-existing anxiety disorder in combination with major mood disorder was associated with a higher risk of suicide attempts in comparison with major mood disorder alone [65-67].

The World Health Organization reported that besides schizophrenia and major mood disorders, minor mental disorders, especially panic disorder and GAD, are also strongly associated with functional disability [68]. Although these disorders usually manifest in a 'mild' clinical picture, this does not imply that they are less incapacitating or burdening, nor that they do not carry a real threat towards physical and mental capabilities. The chronic character of these disorders, their refractory nature and the frustration caused not only to the patient but also to the environment often result in great distress. In addition, if minor mental disorders go untreated or are insufficiently treated, the most frequent long-term complication is the development of major depression [69-74]. In most cases these minor mental disorders seem to be self-restricting in the general population and in the large number of patients treated in primary care. This, however, poses further risks, as the biggest risk for suicide is poorly diagnosed and treated mental disease. Research shows that major depression is a comorbid condition with 'minor' mental disorders in most cases of suicide [6-8,75].

In general, it seems that the same factors and characteristics that determine suicidal behaviour in major depressive patients as well as treatment strategies apply for those suffering from milder forms of depression and anxiety.

## The current status of outcome of intervention concerning suicide

As mentioned earlier, proper diagnosis of major depression or minor mood and anxiety disorders of patients seeking help for psychological problems in general practice and psychiatric care is the most important element in prediction, recognition and treatment of possible suicidal behaviour. It is also important to decide whether the patient's symptoms are the result of unipolar depression or belong to the bipolar spectrum. As antidepressive monotherapy (unprotected by mood stabilisers) can increase or induce risk of suicide in a small part of bipolar patients, this diagnostic distinction is of prime importance After proper diagnosis of depression, it is reasonable to accept that depression is causally related to suicide in a great number of suicidal victims. In this context, it can be expected that successful treatment of depression will lead to a lower risk for suicide [2,8,54,62].

The situation, however, is not always so simple and straightforward. The literature suggests that approximately 75% of depressed suicide victims have a history of previous psychiatric treatment, and 66% have had psychiatric treatment during the previous year. Only 50%, however, were receiving psychiatric treatment at the time of suicide and the rate of specific and adequate antidepressive pharmacotherapy is much lower still [1,6,7,9,12,13,53,56,76]. A key element in the prevention of suicide would be proper recognition of signs and symptoms of approaching suicide. Nearly 20% of suicidal patients visit a physician the actual day they attempt suicide, 40% pay a visit in the preceding week, while 66% contact medical care within 3 months prior to a suicide attempt. These rates are disturbing as they suggest that most depressed suicide victims received neither proper recognition or diagnosis, nor adequate treatment, despite their medical contact. An astonishingly low 3% of suicidal patients received antidepressants in adequate dosages and only 7% percent received psychotherapy [1]. Another study, performed during the pre-SSRI era, found that only 12% of suicide attempters with current major depression received antidepressant pharmacotherapy in adequate doses [13]. There are also significant gender differences in current and previous treatment and suicide methods: males seek and receive treatment less frequently and more commonly use violent suicide methods [1,56,77].

Although minor mood and anxiety disorders constitute a lower risk for suicide, their early detection and appropriate treatment is an important step in suicide prevention, as it substantially decreases the risk of subsequently developing major depression [70,72,78-81] and in this way decreases the risk of further complications, including suicide.

## Conclusion

It is clear that proper and 'aggressive' treatment of major depression aiming at achieving full remission should always be the target, and determines to a large extent whether suicidal behaviour is expressed or not. Any residual symptoms increase the risk of suicide and enhance the burden on patients and their families as well, and lead to the development of a chronic form of the disorder. This chronic condition particularly predisposes patients to demoralisation and the manifestation of suicidal behaviours. It is reasonable to bear in mind that we cannot prevent all suicides. However, earlier recognition and more effective acute and long-term treatment of anxiety and depressive disorders is a key element in suicide prevention [2,58]. The emphasis should be placed on the understanding of the association of suicide with depression, and on the detection and recognition of possible signs of suicidal intent in patients seeking medical help, especially outside psychiatric practice. Today, the vast majority of suicides happen outside the domain psychiatrists see and treat, although victims are likely to suffer from a mental disorder. This is a huge challenge for both medicine and society.
